# Leaf physiological characteristics and grain quality analysis of different types of quinoa-a case study of Shanxi Province, China

**DOI:** 10.3389/fpls.2025.1613459

**Published:** 2025-07-16

**Authors:** Yuzhe Li, Qi Zhang, Jingying Lu, Linzhuan Song, Xinrong Duan, Hongxia Guo, Yan Deng, Li Zhao, Chuangyun Wang

**Affiliations:** ^1^ Agricultural College, Shanxi Agricultural University, Jinzhong, China; ^2^ College of Agronomy, Shanxi Agricultural University, Taiyuan, China

**Keywords:** quinoa, drought-tolerant, senescence characteristics, yield, quality

## Abstract

This study used Q77 as a control to measure the proline content, CAT activity, soluble sugar content and MDA content of three drought-resistant and four non-drought-resistant quinoa varieties at different growth stages, to screen quinoa varieties suitable for planting in alpine regions and for the development of functional foods. Yield and quality were determined after harvesting the grain. The results showed that the yields of Qing1 and Long4 were 27.25% and 21.42% higher than the control, respectively. Both varieties had higher average proline, CAT and soluble sugar contents than the control. Qing1 showed increases of 33.56%, 38.95% and 25.06% respectively, while Long4 showed increases of 29.01%, 40.05% and 22.35%. Their MDA content was 20.6% and 9.41% lower than the control, respectively. Gong8 ranked third in terms of yield (+6.14%), demonstrating strong physiological activity and good quality. B-16 had the best quality: its starch content was approximately 20 percentage points lower than that of corn; its fat content was 10.47 percentage points lower than that of wheat; its protein content was 69.47% higher than that of rice; its dietary fibre content was 48.67 times higher than that of rice; and its essential amino acid content was 1.86 g/100 g higher than that of rice. Correlation analysis revealed that yield was extremely significantly positively correlated with the number of effective branches, main panicle length and 1000-grain weight, with the number of effective branches showing the strongest correlation. Path analysis indicated that MDA content, proline content, CAT activity and soluble sugar content positively affected yield, with proline content contributing the most based on direct path coefficients. In conclusion, Qing1 and Long4 are suitable for large-scale planting in alpine regions, B-16 is primarily suitable for functional food research and development, and Gong8 is suitable for both large-scale planting and functional food development.

## Highlights

- Based on the advantages of different varieties of quinoa, choose the right use.
- The B-16 variety is ideal for functional food research and development.
- Drought-resistant varieties are suitable for cultivation in high-cold regions.
- The effects of malondialdehyde, proline, soluble sugar content, and catalase on yield were positive.
- The proline content directly or indirectly affects the yield of quinoa.

## 1 Introduction

Quinoa (*Chenopodium quinoa* Willd.) is an annual herbaceous plant in the genus Chenopodium of the Amaranthaceae family, originating from the Andean Mountains. It has since been introduced to various regions worldwide and was introduced to China in the 1980s (Cárdenas-Castillo et al., 2021). Due to its cool-loving characteristics and specific requirements for growing environments, most quinoa is planted in cool regions such as Qinghai, Shanxi, Shaanxi, Inner Mongolia, and Gansu.

Due to its unique nutritional composition, quinoa is known as the “mother of grains”. The Food and Agriculture Organization of the United Nations (FAO) has recognized it as the only dicotyledonous plant capable of meeting the basic nutritional needs of humans (De Bock et al., 2021). As quinoa seeds are rich in protein and low in fat, they are sought after by dieters and have high biological value (Pathan et al., 2023). Additionally, quinoa is rich in amino acids and contains various bioactive substances, such as polyphenols, saponins, and flavonoids, which have antioxidant and anti-inflammatory properties (Garcia-Parra et al., 2022; Wang et al., 2024).

Quinoa is a new type of crop that has attracted the attention of agricultural researchers around the world. Yuan et al. (2020) screened 22 high-yielding varieties suitable for planting in Dongchuan, Yunnan Province, from 111 quinoa resources (Yuan et al., 2020); Chen and Liao identified high-protein and low-fat-containing varieties, including Taiqi white quinoa and Shangri-La red quinoa, by studying seven quinoa varieties (Chen and Liao, 2020). These findings provided a new way to approach fat-reduced food and baby food development. Huang et al. (2018) investigated the relationship between agronomic traits and yield in 38 quinoa germplasm resources (Huang et al., 2018). Wang et al. (2022) studied 41 quinoa germplasm resources and concluded that a strong relationship exists between agronomic traits and yield (Wang et al., 2022). Wang conducted a preliminary study on the drought resistance mechanism of quinoa, and the results showed that compared to its other relatives, quinoa has a long root system, high branching, fast growth rate, strong water absorption capacity, a long root system, and strong water absorption capacity; these characteristics contribute to its drought resistance (Wang, 2022). Ma found that by regulating some of its substances, such as proline, soluble sugar, potassium ions, etc., quinoa can improve cellular water-holding capacity to mitigate the damage caused by drought (Ma, 2023). Under drought stress, quinoa may accumulate reactive oxygen species, leading to the loss of dynamic equilibrium, which in turn can damage the biofilm and affect the cellular antioxidant defense system in the plant. The antioxidant defense system of plant cells may resist drought by scavenging free radicals (Hinojosa et al., 2018; Hosseinifard et al., 2022; Ain et al., 2023; Iqbal and Yaning, 2024). Yang investigated changes in enzyme activities by exposing plants to drought stress, and the results showed that the MDA (Malondialdehyd) content and the SOD (SuperoxideDismtase) and CAT (Catalase) activities were directly proportional to the degree of drought stress (Yang, 2021). Zhang found that drought-resistant varieties had higher CAT and SOD activities than drought-sensitive varieties (Zhang, 2011). Ali et al. (2020) showed that the coordination of reservoir-source relationships had a more significant effect on crop yield, the supply of photosynthetically assimilated substances was mainly affected by the source strength, and the yield was mainly affected by the number of panicles, number of branches, and other reservoirs (Ali et al., 2020; Hussain et al., 2020). As most of the energy required for crop growth and metabolism comes from leaf sources, the correlation with yield can be determined by studying the physiological characteristics of quinoa leaves (Asif et al., 2022; Mirsafi et al., 2024).

In recent years, various problems caused by drought have emerged during quinoa cultivation, such as low yield and poor quality. To address these issues, this study takes drought-resistant and non-drought-resistant quinoa varieties as research objects and proposes the hypothesis that drought-resistant varieties exhibit higher proline content, CAT activity, and soluble sugar content, with the enhancement of these activities and contents being positively correlated with improvements in quinoa yield and nutritional quality. The study investigates leaf physiological characteristics, yield, and quality to screen raw materials for functional food research and development, as well as new drought-resistant, high-yielding, and stable-yielding varieties. This aims to provide a theoretical basis and scientific reference for the sustainable development of the quinoa industry, while also offering a new dietary perspective for sub-healthy populations globally.

## 2 Materials and methods

### 2.1 Overview of the test site

The experiment was conducted in 2022 and 2023 at the test site in Jingle County, Xinzhou City, Shanxi Province, which belongs to the north temperate monsoon climate, with an altitude of 1140–2421 m, an average annual frost-free period of 120–135 d, annual precipitation of 380–500 mm, mainly concentrated in July and September, an average annual temperature is 7.2°C, and an average annual sunshine hours of more than 2,600 h. The soil is yellow clay, weakly alkaline, and the 0–20 cm soil layer contains 7.60 g/kg of organic matter and 91 mg/kg of total nitrogen. In 2022, the 0–20 cm layer of the test site contained 7.60 g/kg of organic matter, 91.5 mg/kg of total nitrogen, 128 mg/kg of quick-acting potassium, and 20.23 mg/kg of effective phosphorus; the pH of the soil was 8.14. In 2023, the soil (pH 8.09) contained 7.87 g/kg of organic matter, 95.5 g/kg of total nitrogen, 132 mg/kg of quick-acting potassium, 21.31 mg/kg of effective phosphorus, and 7.87 g/kg of organic matter.

### 2.2 Experimental materials and their handling

Experimental materials: Twelve quinoa varieties were used as experimental materials, and their specific sources are detailed in **Table 1**.

**Table 1:** Experimental cultivars and breeding units.

Cultivars	Breeding units	Growth period length
Qingbaili No.1(Qing1)	Qinghai Academy of Agricultural and Forestry Sciences (Xining,China)	144d
Longli No.4(Long4)	Gansu Academy of Agricultural Sciences (Lanzhou,China)	145d
Gongzha No.8(Gong8)	Tibet Agricultural and Animal Husbandry University (Tibet,China)	143d
Gongzha No.4(Gong4)	Tibet Agricultural and Animal Husbandry University (Tibet,China)	145d
Huaqing No.77(Q77)	Shanxi Huaqing Quinoa products development Co., Ltd (Taiyuan,China)	177d
Huaqing No.60(Q60)	Shanxi Huaqing Quinoa products development Co., Ltd (Taiyuan,China)	164d
Huaqing No.86(Q86)	Shanxi Huaqing Quinoa products development Co., Ltd (Taiyuan,China)	164d
Huaqing No.11(Q11)	Shanxi Huaqing Quinoa products development Co., Ltd (Taiyuan,China)	164d
Huaqing No.93(Q93)	Shanxi Huaqing Quinoa products development Co., Ltd (Taiyuan,China)	164d
Huaqing No.91(Q91)	Shanxi Huaqing Quinoa products development Co., Ltd (Taiyuan,China)	164d
Huaqing No.16(Q16)	Shanxi Huaqing Quinoa products development Co., Ltd (Taiyuan,China)	164d
Black No.16-1(B-16)	Shanxi Huaqing Quinoa products development Co., Ltd (Taiyuan,China)	133d

Q77 is the control group variety.

Handling: After sieving and decontaminating the seeds, 300 intact seeds free of defects and with uniform size were selected, sterilized in a 5% sodium hypochlorite solution for 15 minutes, rinsed three times with sterile distilled water, and placed in sterile Petri dishes; a control group and a drought stress group were set up with three repetitions each, where 7 mL of distilled water was added to the control group and 7 mL of 20% PEG-6000 solution was added to the drought stress group to simulate drought stress conditions (Paradiso et al., 2017; Zhang et al., 2024). Then, 50 seeds were placed in each Petri dish, uniformly lined up with sterilized forceps for counting, and incubated in a light incubator at 20 °C, 60% relative humidity, and a 16 h-8 h light-dark cycle. Germination rates were recorded on days 2, 4, 6, and 8, and germ length, radicle length, fresh weight, and dry weight were measured on day 8. Using the comprehensive ranking method, the membership function method was adopted to standardize each index, converting indices with different units into relative values of 0-1, and then the comprehensive drought resistance score was calculated by arithmetic mean to rank the drought resistance of varieties (**Table 2**). With the local main cultivar Q77 as the control, three drought-tolerant varieties and eight drought-intolerant varieties were preliminarily screened and planted in the field in the same year. Based on the field growth conditions, three drought-tolerant and four drought-intolerant quinoa varieties were determined to study the differences in their leaf senescence characteristics, yield, and quality.

**Table 2:** Membership function value of the drought resistance index and comprehensive evaluation of drought resistance.

Cultivars	Germinability	Germination	Sprout length	Radicel length	Drought tolerance index	Comprehensive score	Rank
Qing1	0.85	0.81	0.17	0.65	0.83	0.662	1
Long4	0.43	0.46	0.62	0.82	0.46	0.558	2
Gong8	0.49	0.54	0.47	0.63	0.46	0.518	3
Q77(CK)	0.48	0.3	0.76	0.48	0.39	0.482	4
Q11	0.31	0.47	0.4	0.76	0.34	0.456	5
Q93	0.34	0.55	0.75	0	0.52	0.432	6
Q91	0.32	0.43	0.3	0.75	0.35	0.43	7
Q16	0.32	0.4	0.31	0.72	0.32	0.414	8
B-16	0.41	0.28	0.28	0.72	0.3	0.398	9
Gong4	0.36	0.26	0.26	0.7	0.3	0.376	10
Q60	0.38	0.25	0.22	0.68	0.28	0.362	11
Q86	0.1	0.3	0.14	0.42	0.3	0.252	12

Each index was standardized to a relative value of 0-1 by the membership function method, so there are no specific units.

### 2.3 Field trial design

The experiment was conducted using a one-way randomized block design. The treatments included eight varieties, three replications, and 24 plots. Each plot had 12 rows; each row was 10 m long, and the spacing between rows was 0.3 m. The area of each plot was 27 m^2,^ and the total experimental area was 648 m².The plots also had artificial cavity nests, and seeds were sown in the first half of June every year. Before sowing through rotary tillage and fertilization integrated machine in rotary tillage, at the same time, a compound fertilizer (N:P_2_O_5_:K_2_O = 22:8:10) 750 kg/hm^2^ was applied, and other management techniques were implemented for increasing the yield, timely weeding, control of pests and diseases, etc. The management measures applied to the experimental plots were identical.

### 2.4 Indicators and methods

#### 2.4.1 Physiological indicators

In the branching, panicle, flowering, grouting, and ripening periods, 15–20 inverted four leaves were collected around 9:00 a.m. to 11:30 a.m. on a sunny day, placed in an ice pot, and transported to the laboratory. The samples were ground, frozen in a tube, and then stored in an ultra-low temperature refrigerator at –80 °C, and kits were used to detect the soluble sugar content, malondialdehyde content, proline content, and CAT activity of the leaves.

##### 2.4.1.1 Determination of CAT enzyme activity

After the quinoa leaves were mashed with liquid nitrogen, 0.1 g was weighed, 1mL of extraction solution was added, homogenized in an ice bath, 8000 g was centrifuged at 4 °C for 10min, the supernatant was taken and placed on ice for testing, 1mL CAT test solution was placed in a 1mL quartz colorimetric dish, then 35 μL samples were added and mixed for 5 s. The absorption value A1 at 240nm and A2 after 1min were measured immediately at room temperature. Calculate ΔA =A1−A2.

CAT activity calculation:

Definition of unit: 1μmol H_2_O_2_ degradation catalyzed per g tissue per minute in the reaction system is defined as a unit of enzyme activity.

The calculation formula for CAT content refers to Equation 1.


(1) $$\begin{array}{c} \text{CAT }\left(\text{U}/\text{g}\right)=\left[\Delta \text{A}\times \text{V inverse total}÷\left(\epsilon \times \text{d}\right)\times 10^{6}\right]\\ ÷\left(\text{W}\times \text{V sample }÷\text{ V sample total}\right)÷\text{T}\\ =678\times \Delta \text{A}÷\text{W} \end{array}$$

Note: V inverse total: total volume of the reaction system, 1.035x10^-3^L; ε: molar absorption coefficient of H_2_O_2_, 43.6 L/mol/cm; d: light diameter of the cupola, 1cm; V sample: add the sample volume, 0.035mL; V sample total: add extraction liquid volume, 1mL; T: reaction time, 1min; W: sample quality, g; 10^6^: unit conversion factor, 1mol=10^6^μmol.

##### 2.4.1.2 Determination of MDA content

After the quinoa leaves are mashed with liquid nitrogen, weigh 0.1g, add 1mL of the extract, homogenate in an ice bath, centrifuge 8000g at 4 °C for 10 minutes, take the supernatant, put it on ice for testing, add reagents according to the sample adding table, keep the mixture in a water bath at 100 °C for 60minutes (cover tightly to prevent water loss), and cool it in an ice bath at room temperature, 10000 g. Centrifuge for 10 min. The absorbance of each sample at 532 nm and 600 nm is measured in the superclear to 1mL glass colorimetric plate, and calculated respectively, ΔA532=A532 determination−A532 blank, ΔA600=A600 determination−A600 blank, ΔA=ΔA532−ΔA600.

The calculation formula for MDA content is referred to Equation 2.


(2) $$\begin{array}{c} \text{MDA content }\left(\text{nmoL}/\text{g mass}\right)=\left[\Delta \text{A}\times \text{V inverse total}÷\left(\epsilon \times \text{d}\right)\times 10^{9}\right]÷(\text{W}\times \text{V sample}\\ ÷\text{V extraction})\times \text{F}=32.258\times \Delta \text{A}÷\text{W} \end{array}$$

Note: V inverse total: total volume of reaction system, 0.001 L; ε: molar absorption coefficient of MDA, 1.55x10^5^ L/mol/cm; V sample: add sample volume, 0.2 mL; d: light diameter of the cupola, 1 cm; V extraction: add the extraction liquid volume, 1 mL; W: sample quality, g; 10^9^: unit conversion factor, 1 mol =10^9^ nmol; F: dilution ratio.

##### 2.4.1.3 Determination of proline content

After the quinoa leaves were mashed with liquid nitrogen, 0.1 g was weighed, 1mL of the extract was added, homogenized in an ice bath, then the leaves were extracted in a boiling water bath under shock for 10 min, centrifuged at 1000g at room temperature for 10 min, the supernatant was obtained, and then cooled to be measured. The standard product was diluted with distilled water to 40, 20, 10, 8, 4, 2, 1, 0.5 μg/mL, and 0.5 mL supernatant, 0.5 mL glacial acetic acid and 0.5 mL reagent in the kit were taken from the measuring tube. Each standard tube is filled with 0.5 ml standard product, 0.5 mL glacial acetic acid and 0.5 mL reagent in the kit. Put 0.5 mL distilled water, 0.5 mL glacial acetic acid and 0.5 mL reagent in the kit in a blank tube. After mixing, cover tightly, wrap the sealing film, keep warm in boiling water bath for 30 min, shake once every 10 min, after cooling, compare color at 520 nm wavelength, record light absorption value A determination tube, A standard tube and A blank tube, calculate ΔA=A determination tube −A blank, ΔA standard =A standard −A blank. Draw A curve with standard solution as the abscissa and standard ΔA as the ordinate to get the linear regression equation y=kx+b, and substitute ΔA into the equation to get x (μg/ml). The calculation formula for proline content is referred to Equation 3.


(3) $$\begin{array}{c} \text{Proline content calculation}:\text{Pro content }\left(\text{μg}/\text{mL}\right)\\ =\text{x}\times \text{V}÷\text{W}=\text{x}÷\text{W} \end{array}$$

Note: V extraction: add extraction liquid volume, 1 mL; W: sample quality, g.

##### 2.4.1.4 Determination of soluble sugar content

After the quinoa leaves were mashed with liquid nitrogen, 0.1 g was weighed, 1mL of distilled water was added to the homogenized pulp, and then poured into a covered centrifugal tube, boiling water was bathed for 10min, and 8000 g was cooled and centrifuged at room temperature for 10min. The supernatant was placed in a 10ml test tube, filled with distilled water to 10mL, and shaken well for use. Dilute the standard with distilled water to 0.2, 0.1, 0.05, 0.025, 0.0125, 0.00625 mg/mL. 400 μL distillated water, 100 μL working solution and 1000μL concentrated sulfuric acid were added into the blank tube. Measuring tubes were filled with 200 μL sample, 200 μL distillated water, 100 μL working solution and 1000 μL concentrated sulfuric acid. Add 200μL standard, 200 μL distilled water, 100 μL working liquid, 1000 μL concentrated sulfuric acid into standard tube. Mixed well, placed in 95°C water bath for 10 min, cooled and measured at 620 nm light absorption value, respectively recorded as A blank tube, A determination tube, A standard tube, calculate ΔA=A determination tube −A blank tube. According to the standard tube concentration (x, mg/ml) and absorbance ΔA standards, A standard curve is established, and according to the standard curve, ΔA (y, ΔA) is substituted into the formula to calculate the sample concentration (x, mg/mL). The calculation formula for soluble sugar content is referred to Equation 4.


(4) $$\begin{array}{c} \text{Calculation of Soluble Sugar content}:\text{Soluble Sugar }\left(\text{mg}/\text{g masst}\right)\\ =10\times \text{x}÷\text{W} \end{array}$$

Note: W: sample quality.

Tissuelyser-96, the automatic sample rapid grinding instrument of Shanghai Jingxin ((Shanghai, China) Technology Co., LTD., was used for grinding; Use the electronic balance HZT-A+200 of Huazhi (China) Electronic Technology Co., LTD to weigh; Centrifuge 5810 R, an eppendorf (Hamburg, Germany) centrifuge, is used for centrifuge. The UV-visible spectrophotometer UV9100D S/N:1905UV1849 of Beijing Lebertai Scientific Instrument Co., LTD (Beijing, China) was used for the determination. The biochemical reagent kit provided by Beijing Solarbio Science & Technology Co., Ltd. (Beijing, China) was used to detect.

#### 2.4.2 Determination of seed quality

After shelling the seeds, the seeds without pests, diseases, and mechanical damage were selected, and the national standard method was used to determine the relevant qualities. Protein: GB 5009.5–2016 the first method; Fat: GB 5009.6–2016 the second method; Dietary fiber: GB 5009.88-2014; Starch: GB 5009.9–2016 the second method; Amino acids: GB 5009.124-2016.

#### 2.4.3 Yield and yield composition

The quinoa seeds were harvested when the stalks turned yellow, and 80% of the leaves turned yellow. Ten plants were selected at fixed points for sampling, the length of their panicles and the number of effective branches were determined, and they were dried and threshed. Finally, they were weighed, and the weight of 1,000 grains and yield were determined.

### 2.5 Data processing

Data processing was performed using Microsoft Excel 2016. Statistical analyses for examining differences were conducted with SPSS 16.0 software (SPSS Inc., Chicago, Illinois, USA). Analysis of variance (ANOVA) and least significant difference (LSD) calculations were employed to identify statistically significant differences (p< 0.05). Following the analyses, data visualization was carried out through graphing.

## 3 Results and analysis

### 3.1 Differences in leaf senescence characteristics of different quinoa types

#### 3.1.1 MDA content of quinoa leaves

**Figure 1:**
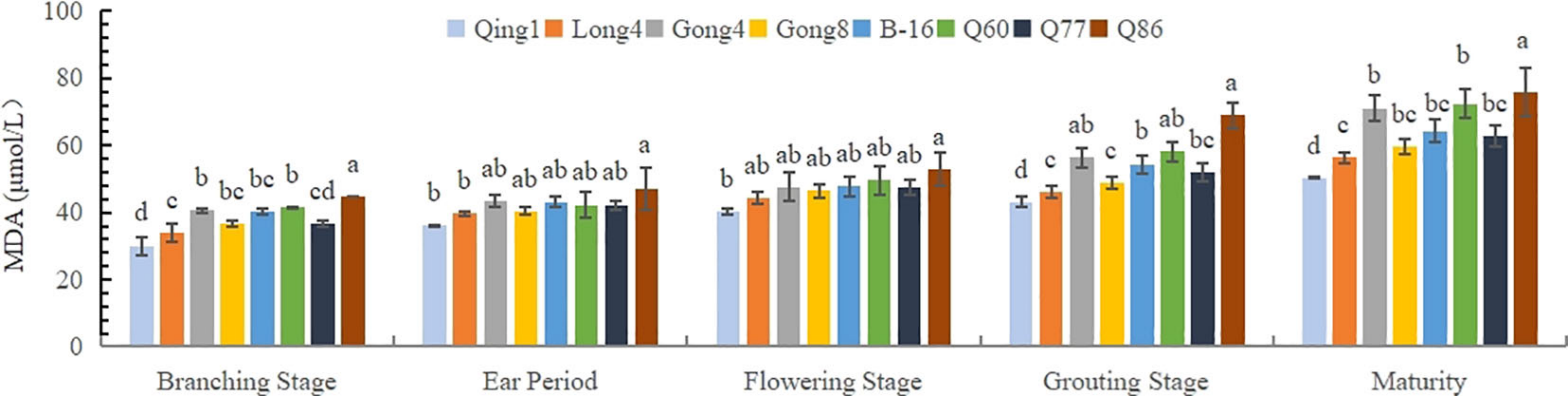
Changes in the MDA content in quinoa leaves. Different letters in each column indicate significant cant differences at p < 0.05.

Malondialdehyde (MDA) can reflect the degree of lipid peroxidation in the body and indirectly indicate the degree of cell damage. As shown in **Figure 1**, the MDA content in the leaves of different quinoa varieties gradually increased with the progression of the growth period, reaching the maximum at maturity. At each growth stage, the MDA contents of Qingbaili No. 1 (39.99 μmol·L^-1^), Longli No. 4 (44.08 μmol·L^-1^), and Gongzha No. 8 (46.35 μmol·L^-1^) were lower than those of No.77(CK) (48.23 μmol·L^-1^), indicating lower levels of lipid peroxidation and less plasma membrane damage. The MDA contents of Gongzha No. 4, Hei 16-1, No. 60, and No. 86 were 7.47%, 3.7%, 9.51%, and 20.01% higher than those of the control, respectively, suggesting greater degrees of cell membrane damage. Notably, at maturity, the differences in MDA content among treatments were the most pronounced.

#### 3.1.2 CAT activity of quinoa leaves

**Figure 2:**
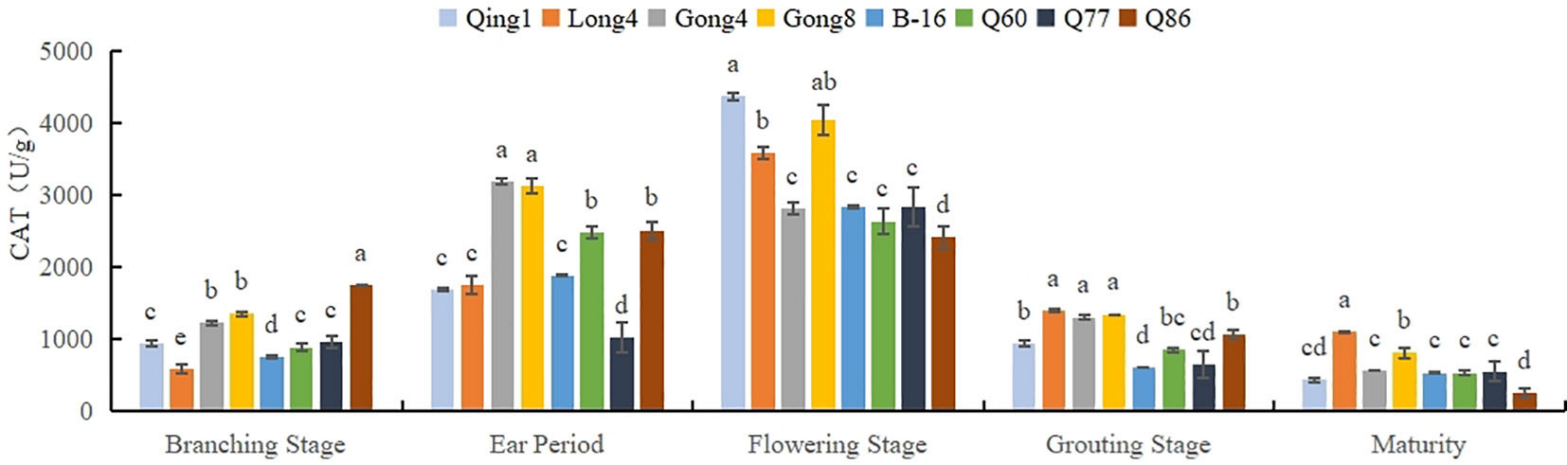
Changes in the CAT activity of quinoa leaves. Different letters in each column indicate significant cant differences at p < 0.05.

As shown in **Figure 2**, the CAT activity in quinoa leaves exhibited a trend of first increasing and then decreasing with the progression of the growth period, reaching a peak at the flowering stage, followed by a rapid decline. The highest CAT activity at the flowering stage was observed in Qingbaili No. 1, reaching 4367.52 U/g, while the lowest was in No. 86, at 2411.17 U/g. Overall, the average CAT activities of Qingbaili No. 1, Longli No. 4, and Gongzha No. 8 across all growth stages were 1674.67 U/g, 1687.91 U/g, and 2136.23 U/g, respectively, all of which were higher than that of the control (CK, 1205.26 U/g).

#### 3.1.3 Proline content of quinoa leaves

**Figure 3:**
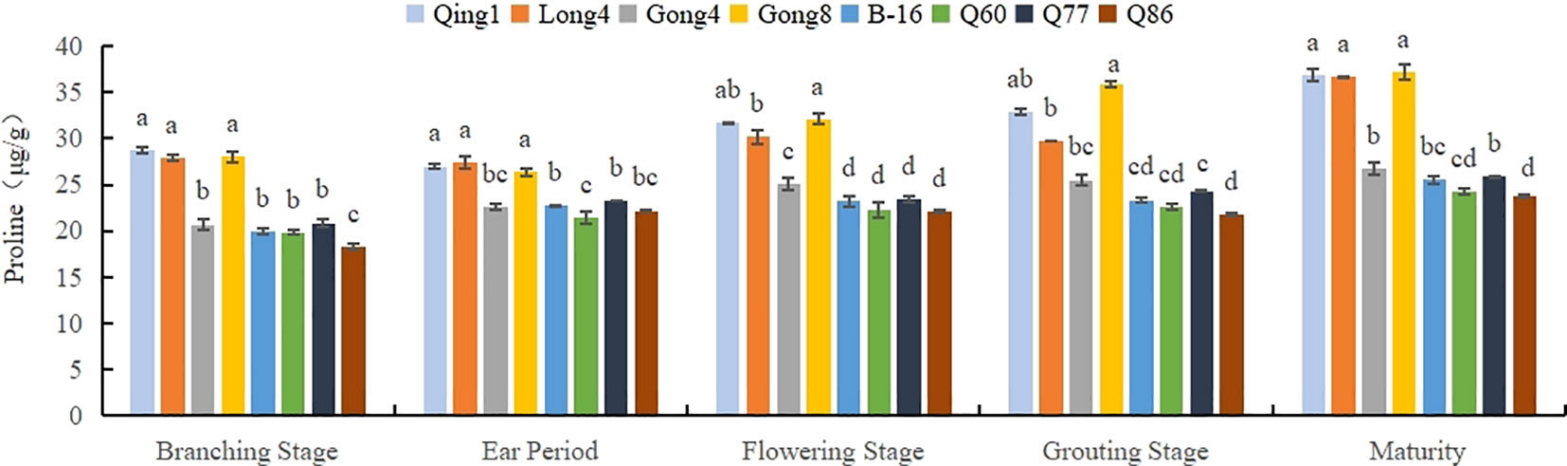
Changes in the Proline Content in Quinoa Leaves Note: Different letters in each column indicate significant cant differences at p < 0.05.

As shown in **Figure 3**, with the progression of the growth period, the proline content in the leaves of these eight different quinoa varieties increased to varying degrees. Among them, the proline accumulation in Qingbaili No. 1 (31.44 μg/g), Longli No. 4 (30.37 μg/g), and Gongzha No. 8 (31.91 μg/g) was significantly higher than that in the control (CK, 23.54 μg/g). The proline contents in No. 60, No. 86, Hei 16-1, and Gongzha No. 4 were 6.17%, 8.17%, 2.51%, and 0.25% lower than those in the CK, respectively. The order of proline content was Qingbaili No. 1 > Longli No. 4 > Gongzha No. 8 > Q77 (control) > Hei 16-1 > Gongzha No. 4 > No. 60 > No. 86, indicating that the proline content in drought-tolerant varieties was significantly higher than that in drought-sensitive varieties.

#### 3.1.4 Soluble sugar content

**Figure 4:**
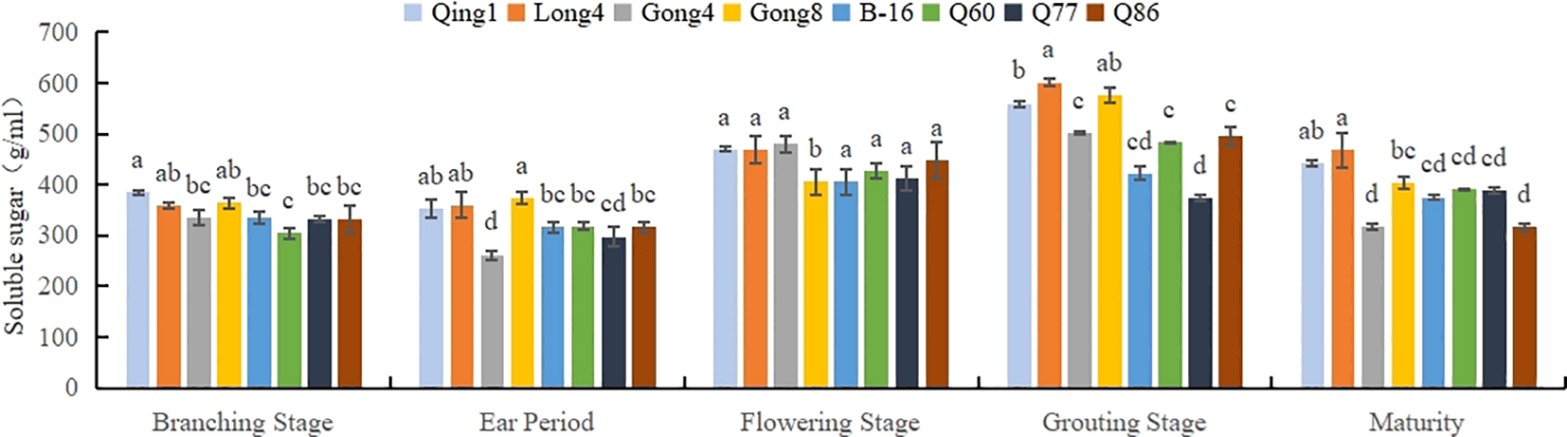
Changes in the soluble sugar content in quinoa leaves. Different letters in each column indicate significant cant differences at p < 0.05.

As can be seen in **Figure 4**, the soluble sugar content in the leaves first increased and then decreased as the reproductive period progressed; the maximum value was recorded during the filling period. The accumulation of soluble sugar in different varieties of quinoa was different; specifically, Qing 1 had the highest content throughout the period, with an average value of 451.53 mg/g. Long quinoa No. 4 had the second highest content (441.73 mg/g), and B-16 had the lowest content of soluble sugar (370.67 mg/g). The soluble sugar content in different quinoa varieties followed the order Qing 1 > Long 4 > Gong 8 > Q86 > Q60 > B-16.

### 3.2 Analysis of the yield and yield component indicators

The yield performance of different varieties of quinoa was different (**Table 3**). Qing 1, Long 4 and Gong 8 yields were 29.78%, 25.09%, and 6.14% higher than those of Q77, respectively. Qing 1 had the highest yield of 2940.25 kg/hm^2^; its effective branching number, main panicle length, and thousand-grain weight were also the highest. The Q86 variety had the lowest yield (1965.21 kg/hm^2^), the lowest effective number of branches, and the shortest main panicle length. Its yield was 17.57% lower than that of the control. The production potential of quinoa is synergistically affected by the effective number of branches and the panicle length. The 1000-grain weigh of Qing 1, Long 4, and Gong 8 was 12.38%, 8.50%, and 3.16% higher than that of Q77, respectively, and B-16, Gong 4, Q60 and Q86 were 2.74%, 3.26%, 17.71% and 52.03% lower than those of the control (Q77), respectively. The thousand-grain weights of the varieties followed the order Qing 1 > Long 4 > Gong 8 > Q77 > Black-16 >> Gong 4 > 60 > Q60 > Q86.

**Table 3:** The yield composition of different drought-tolerant quinoa varieties.

Cultivars	Plant branch (cm)	Panicle length (cm)	1000-grain weight (g)	Yield (kg/hm^2^)
Qing1	24.67±0.33a	57.67±5.36a	4.63±0.74a	2940.25±12.11a
Long4	18.00±1.15bc	54.67±4.67a	4.4±0.927ab	2805.48±10.09a
Gong8	19.67±0.88ab	45.67±4.41abc	4.25±1.03ab	2452.46±23.16b
Q77(CK)	17.67±4.81bc	47.00±1.00ab	4.12±0.87ab	2310.53±22.18bc
B-16	15.33±1.86bc	52.67±1.86ab	4.01±1.13bc	2265.56±18.79bc
G0ng4	15.67±2.19bc	40.33±6.94bc	3.99±1.04bc	2242.78±14.08bc
Q60	14.67±0.88bc	34.00±1.15cd	3.50±0.78c	2077.50±12.56cd
Q86	12.33±0.67c	27.00±1.00d	2.71±0.69d	1965.21±33.79d

The data in the table represent the mean ± standard deviation. The lowercase letters in each column with different superscripts indicate differences between various treatments at the 0.05 level.

The results of the correlation analysis (**Table 4**) showed that the number of branches, the length of the main panicle, and the thousand-grain weight were positively correlated with yield. The effective number of branches had the strongest correlation with yield, followed by the thousand-grain weight, and finally, the length of the main panicle. The length of the main panicle and the effective number of branches were significantly correlated with each other, with a correlation coefficient of 0.887. The three factors complemented each other and worked together to improve the yield. The yield could be improved by adopting measures to increase the length of the main panicle, the effective number of branches, and the thousand-grain weight.

**Table 4:** The yield correlation coefficient of different drought-tolerant quinoa varieties.

Yield Components	Plant branch	Panicle length	1000-grain weight	yield
Plant branch	1			
Panicle length(cm)	0.767^*^	1		
1000-grain weight(g)	0.832^*^	0.925^**^	1	
yield(kg/hm^2^)	0.887^**^	0.866^**^	0.867^**^	1

“*” indicates significant correlation (*P*<0.05); “**” indicates extremely significant (*P*<0.01).

### 3.3 Analysis of the differences in seed quality

#### 3.3.1 Basic nutritional composition

**Figure 5:**
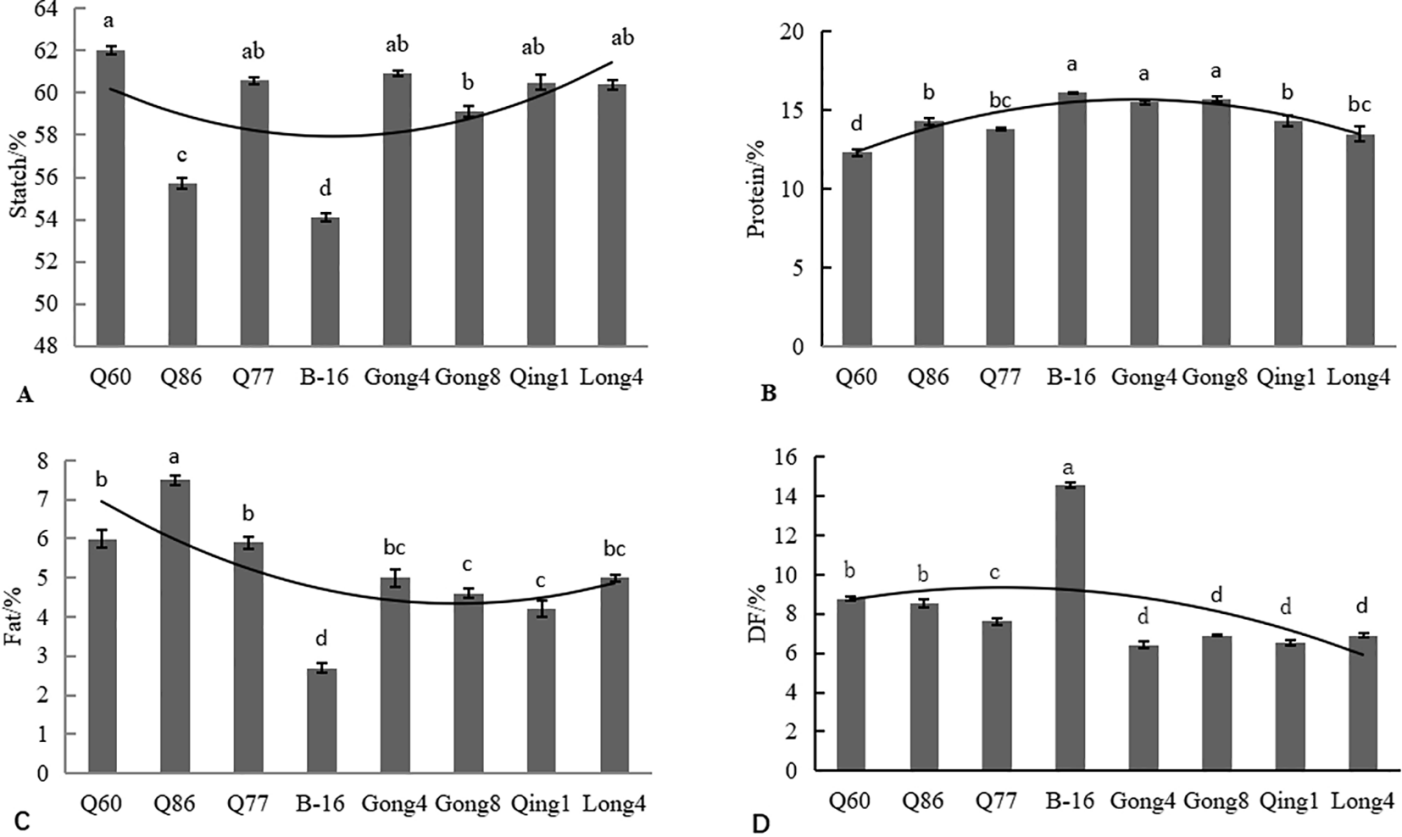
Analysis of nutritional components of different quinoa cultivars, including starch **(A)**, protein **(B)**, fat **(C)**, and dietary fiber **(D)**. Different letters in each column indicate significant differences at the p < 0.05 level.

Grains are an important component of human staple food and can provide a large amount of energy. Quinoa is rich in more dietary fibers and proteins, but poor in fats compared to other major grain crops, such as rice, wheat, and corn. As can be seen from **Figure 5A**, the starch content of all eight quinoa varieties ranged from 54.10% to 62.00%, and the contents, in descending order, were as follows: Q60 (62.00%) > Gong 4 (60.90%) > Q77 (60.60%) > Qing 1 (60.50%) > Long 4 (60.40%) > Gong 8 (59.10%) > Q86 (55.70%) > B-16 (54.10%).

The protein content of the eight quinoa varieties ranged from 12.3% to 16.1% (**Figure 5B**). The B-16 variety had the highest protein content (16.1%), and Q60 had the lowest protein content. All eight quinoa resources had higher protein content than common cereals, including maize (10.2% to 12.8%), wheat (11.7%), rice (7.6% to 9.2%), and oats (10.5% to 11.7%).

The fat content of the eight quinoa materials ranged from 2.70% to 7.50% (**Figure 5C**). The Q86 variety had the highest fat content of 7.50%, and B-16 had the lowest fat content of 2.70%. The quinoa materials selected in this study had a lower fat content than wheat (13.17%) and rice (11.70%). The fat content of these varieties was similar to those of corn (3.80%) and millet (1.70%). To summarize, we found that quinoa had a low fat content and may be used to develop low-fat foods that are suitable for weight loss.

The content of dietary fiber in the eight quinoa materials ranged from 6.42% to 14.60% (**Figure 5D**). Gong 4 had the lowest dietary fiber content of 6.42%, and B-16 had the highest content of 14.60%, which was 2.3 times higher than that of Gong 4. The dietary fiber content of the varieties followed the order: B-16 (14.60%)>Q60 (8.78%) > Q86 (8.57%) > Q77 > Gong 8 (6.92%) > Long 4 (6.90%) > Qing 1 (6.52%)>Gong 4 (6.42%).

#### 3.3.2 Amino acid content

A complete range of amino acids was present in the eight quinoa materials. As indicated in **Table 5**, the eight quinoa materials encompassed the complete range of amino acids. Among them, B-16 had the highest total amino acid content, and the size of the total amino acid content followed the order B-16 > Gong 8 > Gong 4 > Q86 > Qing 1 > Q77 > Long 4 > Q60. The content of leucine was the highest (7.23%) among all essential amino acids. A higher leucine content was recorded in Black 16-1, Gong 4, and Gong 8. The homologous amino acid content of different quinoa materials varied significantly, and the results of a comprehensive analysis showed that B-16 had the optimal amino acid content, with its leucine (1.00%), isoleucine (0.60%), histidine (0.64%), phenylalanine (0.61%), and lysine (0.84%) contents at the top of the list of essential amino acids. Its alanine content was 1.18 times higher than that of the control (Q77; 0.57%), and histidine content was 1.28 times that of the control (0.50%). Additionally, the amino acid contents of Gong 4 and Gong 8 were also higher.

**Table 5:** Types and contents of amino of quinoa accessions %.

Cultivars	Met	Val	Lys	Ile	Phe	Leu	His	Thr
Qing1	0.15±0.03ab	0.66±0.14c	0.8±0.13bc	0.56±0.12abc	0.56±0.11abc	0.91±0.31b	0.26±0.09d	0.48±0.10abc
Long4	0.16±0.06a	0.58±0.12d	0.74±0.09d	0.51±0.18cd	0.52±0.16cd	0.84±0.15cd	0.32±0.07a	0.46±0.16bc
Gong8	0.16±0.08a	0.62±0.11b	0.84±0.15a	0.58±0.22ab	0.60±0.09a	0.94±0.21b	0.32±0.15a	0.52±0.12a
Q77	0.15±0.03ab	0.63±0.11bc	0.78±0.07c	0.54±0.08bc	0.54±0.11bcd	0.89±0.17bc	0.31±0.09ab	0.43±0.12cd
B-16	0.11±002d	0.72±0.13a	0.84±0.14a	0.6±0.02a	0.61±0.05a	1.00±0.07a	0.28±0.08c	0.49±0.16ab
Gong4	0.14±0.04bc	0.64±0.10bc	0.83±0.17ab	0.58±0.16ab	0.58±0.10ab	0.94±0.13b	0.30±0.08b	0.50±0.13ab
Q60	0.13±0.02c	0.56±0.02d	0.72±0.23d	0.48±0.12d	0.49±0.08d	0.80±0.16d	0.30±0.06b	0.40±0.17d
Q86	0.14±0.06bc	0.63±0.15bc	0.84±0.05a	0.55±0.07abc	0.56±0.13abc	0.91±0.23b	0.30±0.13b	0.50±0.13ab
	Ala	Ser	Asp	Gly	Arg	Tyr	Pro	Glu
Qing1	0.59±0.10abc	0.58±0.05bc	1.24±0.14c	0.79±0.11a	1.06±0.24ab	0.26±0.07d	0.52±0.09ab	2.06±0.08b
Long4	0.56±0.09c	0.54±0.04cd	1.15±0.21c	0.70±0.20cd	1.04±0.22abc	0.32±0.10a	0.46±0.08bcd	1.92±0.10c
Gong8	0.61±0.11ab	0.60±0.03ab	1.32±0.12c	0.79±0.13a	1.14±0.12a	0.32±0.08a	0.55±0.07ab	2.22±0.08a
Q77	0.57±0.09bc	0.58±0.08bc	1.20±0.09cd	0.72±0.16bc	0.86±0.11cd	0.31±0.11ab	0.41±0.12cd	1.87±0.12c
B-16	0.67±0.03a	0.65±0.07a	1.28±0.13c	0.74±0.15b	0.90±0.22bcd	0.28±0.09c	0.50±0.07abc	2.09±0.08ab
Gong4	0.60±0.04ab	0.60±0.04ab	1.30±0.20c	0.78±0.21a	1.18±0.31a	0.30±0.12b	0.56±0.15a	2.09±0.11ab
Q60	0.52±0.03d	0.52±0.12d	1.07±0.25d	0.67±0.06d	0.78±0.21d	0.30±0.08b	0.38±0.09d	1.68±0.07d
Q86	0.59±0.02abc	0.58±0.10bc	1.22±0.16d	0.78±0.12a	0.98±0.09ab	0.30±0.06b	0.56±0.10a	2.10±0.14ab

The data in the table represent the mean ± standard deviation. The lowercase letters in each column with different superscripts indicate differences between various treatments at the 0.05 level.

### 3.4 Pass-through analysis between physiological indices and yield during the irrigation period for different quinoa varieties

To determine the effect of physiological indicators on yield, a path analysis was conducted between physiological indicators and yield during the irrigation period. X_1_, X_2_, X_3_, and X_4_ represented the MDA content, proline content, CAT activity, and soluble sugar content, respectively. The effects of MDA content, proline content, CAT activity, and soluble sugar content on yield were all positive (**Table 6**), and the direct path coefficients showed that the positive contribution followed the order proline content (X_2_) > CAT activity (X_3_) > MDA content (X_1_) > soluble sugar content (X_4_). The indirect throughput coefficient showed that the size of the indirect effect of each physiological index on yield was different, but all of them played a positive role. The positive effect of proline content (X_2_) → soluble sugar content (X_4_) → yield (Y) was the largest (0.903); proline content (X_2_)→CAT activity (X_3_)→yield (Y) had the next largest positive effect (0.671). The synthesis showed that the proline content could directly or indirectly affect the yield of quinoa.

**Table 6:** Path analysis of the relationship between physiological indexes and yield of different drought tolerant quinoa cultivars at filling stage.

Character	Direct path coefficient	Indirect path coefficient			
		X_1_→Y	X_2_→Y	X_3_→Y	X_4_→Y
X_1_	0.272		0.002	0.075	0.024
X_2_	1.258	0.01		0.671	0.903
X_3_	0.635	0.176	0.338		0.533
X_4_	0.101	0.009	0.073	0.085	

## 4 Discussion

Plants can effectively prevent the damage caused by free radicals by activating the protective enzyme system under adversity, and the degree of mobilization of protective enzymes varies among plants with different levels of drought tolerance (Omarova et al., 2020). Under drought stress, mainly, the cytoplasmic membrane system of plants is injured (Yang et al., 2021). MDA is often used as an important marker to determine the degree of membrane lipid peroxidation, which damages the plasma membrane of quinoa leaves under drought conditions. The results of this study were similar to those of previous studies (Ling et al., 2015; Sadak et al., 2019; Eskikoy and Kutlu, 2024), the MDA content of the leaves of different quinoa varieties increased with the advancement of the reproductive period and reached a maximum value at the maturity stage. The MDA content of drought-tolerant varieties was lower than that of the drought-intolerant varieties (Chao et al., 2018; Lin and Chao, 2021; Benaffari et al., 2022).

Through the integrated analysis of drought tolerance indices and yield data, it is evident that drought-tolerant varieties like Qing 1 maintain superior growth performance (more branches, longer panicles) under drought stress, ensuring grain development (high 1000-grain weight) and ultimately achieving higher yields. This pattern clearly demonstrates that drought tolerance acts as a core determinant for ensuring yield stability in arid environments, with drought-tolerant varieties exhibiting significant yield advantages. These findings provide direct empirical support for breeding drought-resistant quinoa varieties and optimizing planting strategies in arid regions.

Plants use antioxidant enzymes to protect against external stress. Protective enzymes can decrease the rate of plasma membrane oxidation by scavenging free radicals, thus enhancing drought tolerance (Farooq et al., 2009; Dong et al., 2019). In this study, CAT activity first increased and then decreased. The antioxidant capacity of different varieties of quinoa was different, and the CAT vigor of drought-tolerant quinoa varieties was higher than that of drought-sensitive varieties in each period, which indicated that quinoa scavenges excess reactive oxygen species by increasing CAT activity, thus reducing the damage to itself. As the reproductive period advanced, the water content of the leaves decreased, the accumulation of free radicals increased, the protease was damaged, and enzyme activity decreased. Hou, Liyuan also showed that quinoa with high CAT activity has high antioxidant properties and drought tolerance, and the CAT activity of quinoa leaves first increased and then decreased as the fertility process advanced (Farooq et al., 2009).

The degree of drought tolerance in plants is positively correlated with the accumulation of proline, i.e., it increases with the degree of drought. Proline acts as a cellular regulator to increase the concentration of the cytosol and decrease the cellular osmotic potential for water retention. In this study, we found that the proline content in the leaves of drought-tolerant varieties was considerably higher than that of CK, which matched the results of another study (Toscano et al., 2016). Additionally, the proline content increased greatly during the flowering and grouting stages, which indicated that the effect of proline was higher in the later stages of quinoa fertility than in the earlier stages, and the difference in the proline content of quinoa varieties with different drought resistance was more prominent.

Under stress conditions, plants maintain water balance, increase cellular osmotic pressure, and protect cell membranes through soluble sugars. Therefore, soluble sugar content in plants serves as a critical indicator for evaluating plant drought resistance. During the grain-filling stage, soluble sugar content reaches its peak and gradually decreases thereafter. The peak of soluble sugar content during the grain-filling stage indicates that the maturation of quinoa grains consumes substantial carbohydrates. In the early grain-filling stage, leaves still exhibit strong photosynthetic capacity, but grains are not fully developed, and the sugar transport rate has not yet reached its maximum. Consequently, partial sugars remain in the leaves, forming the content peak. As the grain-filling process progresses, the demand from grains exceeds the synthetic capacity of leaves, leading to a decrease in soluble sugar content. This was similar to the findings of Chun et al. (2018), who showed that the soluble sugar content in the leaves first increased and then decreased as the reproductive stage progressed; the highest soluble sugar content was recorded at the filling stage (Wang et al., 2022).

The results of the correlation analysis between yield and panicle showed that the effective branching number had the closest correlation with yield. Based on this finding, the yield might be increased by increasing the effective branching number. The yields of the quinoa varieties in this study were lower than those reported in Qinghai, Gansu, Hebei, and Tibet (Ogungbenle, 2003) and higher than those reported in Yunnan, which might be related to the differences between planting sites, planting densities, natural conditions, and resources available for testing. The yield of the different varieties followed the order Qingbai quinoa1 > Long quinoa4 > Gongzha 8 > Q77 > Black 16-1 > Gongzha 4 > Q60 > Q86. Specifically, Qing 1 and Long 4 had the highest yields of 2,940.85 kg/hm^2^ and 2,805.48 kg/hm^2^, respectively, which indicated that drought-resistant quinoa had higher yields. These results also indicated that the two varieties were adapted to the local ecological environment and could be planted as the main varieties. Drought-sensitive quinoa had lower yields; Q60, Q86, and B-16 had 0.11, 0.18, and 0.02 times lower yield than CK (Q77), respectively. These findings were similar to those of Hao et al. (2022), who showed that the yield correlation coefficients of drought-tolerant varieties were higher than those of drought-sensitive varieties.

Ogungbenle (Hao et al., 2022) showed that quinoa has a higher nutrient content than common grain crops and is a low-calorie and high-protein food, which was also found in this study. The average content of protein, fat, dietary fiber, and starch of the eight quinoa varieties in this study was 14.61%, 5.15%, 8.34%, and 58.99%, respectively. The protein content was higher than that of common cereal crops, such as corn (10.20–12.80%), wheat (9.80–11.70%), rice (7.60–9.20%), and oats (10.50–11.70%), while the fat content was lower and the dietary fiber was higher, especially B-16 was the most prominent. Due to its low fat and high protein content, quinoa is favored by many fitness professionals.

No significant difference in the amino acid content was found in different drought-tolerant quinoa varieties, and the content was higher than the values reported in other studies (Wen et al., 2024) The first limiting amino acid in all eight quinoa materials in this study was tyrosine, which matched the results of the study by Lv et al. (2022) Tryptophan was not detected probably because it was dissolved in water during the assay. Some studies have shown that lysine is the first limiting amino acid in most grains (Yang et al., 2018), whereas quinoa has a high lysine content, accounting for 19.70% of the total essential amino acids. Thus, quinoa is more suitable to be developed and utilized as a nutritional supplement compared to common grains. B-16 has the highest lysine content, and the contents of histidine, alanine, serine, leucine, isoleucine, and phenylalanine are higher in B-16 than in other genotypes of quinoa. Overall, B-16 is the healthiest among the eight quinoa resources.

## 5 Conclusion

All selected varieties can be planted in Shanxi Province, but because they have different characteristics, their uses are different. Qingbaili No.1 showed strong physiological activity, excellent seed quality, and the highest yield, which was 27.25% higher than the yield of the control. Longli No.4 had the second-highest yield, which was higher than the yield of CK. It also showed strong physiological activity and the best quality. Therefore, Qingbaili No.1 and Longli No.4 are suitable for planting in large alpine areas in the north-central part of Shanxi Province. B-16 had lower fat and starch content, higher dietary fiber and protein content, and high content of various species of amino acids. Thus, this variety is ideal for the research and development of functional foods. The yield, physiological activity, and the number of amino acid species of Gongzha 8 were similar to those of CK. Gongzha 8 can be planted in large areas to enhance economic benefits and can also be used as a raw material for functional food development. The relationship between the yield, panicles, and physiological activity during the filling period showed that the effective branching number, main panicle length, thousand-grain weight, MDA content, proline content, CAT activity, and soluble sugar content had positive effects on yield. Specifically, the effective branching number and proline content had the greatest effect on yield.

## Data Availability

The original contributions presented in the study are included in the article/supplementary material. Further inquiries can be directed to the corresponding authors.
